# Supplementary material for: A Critical View of the Application of the APEX Software (Aqueous Photochemistry of Environmentally-Occurring Xenobiotics) to Predict Photoreaction Kinetics in Surface Freshwaters
Source: Molecules. 2019 Dec 18;25(1):9. doi: 10.3390/molecules25010009 (PMC7017383; doi:10.3390/molecules25010009)
Supplement: Supplementary file 1 [file molecules-25-00009-s001.zip › 3.2.4_gcc-4.4.0.zip!/3.2.4_gcc-4.4.0/3.2.4_gcc-4.4.0/doc/octave/html/interpreter/Looping-Over-Structure-Elements.html]

Looping Over Structure Elements - Untitled


Up: The <code>for</code> Statement


---

#### 10.5.1 Looping Over Structure Elements

A special form of the `for` statement allows you to loop over all
the elements of a structure:

```
     for [ val, key ] = expression
       body
     endfor
```

In this form of the `for` statement, the value of expression
must be a structure. If it is, key and val are set to the
name of the element and the corresponding value in turn, until there are
no more elements. For example,

```
     x.a = 1
     x.b = [1, 2; 3, 4]
     x.c = "string"
     for [val, key] = x
       key
       val
     endfor
     
          -| key = a
          -| val = 1
          -| key = b
          -| val =
          -|
          -|   1  2
          -|   3  4
          -|
          -| key = c
          -| val = string
```

The elements are not accessed in any particular order. If you need to
cycle through the list in a particular way, you will have to use the
function `fieldnames` and sort the list yourself.

The key variable may also be omitted. If it is, the brackets are
also optional. This is useful for cycling through the values of all the
structure elements when the names of the elements do not need to be
known.
